# How an aging society affects the economic costs of inactivity in Germany: empirical evidence and projections

**DOI:** 10.1186/s11556-017-0187-1

**Published:** 2017-10-17

**Authors:** Sören Dallmeyer, Pamela Wicker, Christoph Breuer

**Affiliations:** Department of Sport Economics and Sport Management, German Sport University Cologne, Am Sportpark Muengersdorf 6, 50933 Cologne, Germany

**Keywords:** Costs of inactivity, Aging societies, Projections, Physical activity, Public health

## Abstract

**Background:**

Aging societies represent a major challenge for health care systems all over the world. As older people tend to be more physically inactive, economic costs of inactivity are likely to increase notably. The present study aims to investigate this relationship between an aging society and economic costs of inactivity using the example of Germany.

**Methods:**

Using data from the German Socio-Economic Panel, this study applied the comparative risk assessment method developed by the WHO to estimate the direct costs of inactivity for the period 2001–2013 differentiated by gender-specific age-groups (15–29; 30–44; 45–64; 65+). Based on population statistics predicting the aging of the German population for the years 2014–2060, this research projects the development of future costs of inactivity and potential effects of interventions promoting physical activity among the German population.

**Results:**

The results reveal an increase in the level of physical activity during the observed period (2001–2013) which compensated the negative effect of aging and resulted in a decline of inactivity costs. The projections for the years 2014–2060 indicate a constant increase in direct per capita costs until 2060 because of an aging society. Scenarios indicating how a short-term reduction of physical inactivity impacts costs of inactivity reveal the crucial role of the oldest age-group in this context.

**Conclusion:**

The findings indicate that the aging of the German population demands further actions and initiatives to promote physical activity, especially for the oldest age-group.

## Background

Demographic trends projected over the next decades indicate a considerable aging of the world’s population [1]. Notable changes in societies’ age structure caused by expected dynamics in fertility, life expectancy, and migration will challenge public health systems from countries all over the world [2]. For example, Richardson and Robertson [3] predicted an annual increase in total health spending of 0.6% from 1995 to 2051 caused by the aging of the Australian population. For the United States, Alemayehu and Warner [4] projected an increase in total per capita health costs of 20% due to aging from 2000 to 2030 with an annual increase of 0.6% and the European Commission [5] stated that only because of demographic changes public health care expenditure in the European Union would increase from 6.9% to 8% of national gross domestic product (GDP) between 2013 and 2060. While the above studies have looked at an aggregated effect of aging, another stream of research has conducted a disaggregated analysis by investigating the incidence of various diseases, such as cardiovascular diseases, pelvic floor disorders, and obesity and forecasting the respective costs over the next decades [6–8]. The results of this research have indicated that aging affects some clinical conditions more than others, especially heart and vascular diseases [9].

As a consequence, governments developed strategies to counteract the alarming development for certain clinical conditions. To that end, physical activity represents an effective mechanism. Research has identified particularly older people to be physically inactive [10]. While there appears no linear relationship between age and physical activity [11], people tend to reduce their level of physical activity from a certain age onwards [12]. Given that age-appropriate physical activity benefits the elderly in numerous ways, such as reduced incidences of high blood pressure, stroke, Type 2 diabetes, and cancer [13], high rates of inactivity can be considered a major driver of age-related health care costs. Therefore, governments have tried to promote physical activity among older population groups, for example through campaigns [14], provision of specific facilities [15], or activity-friendly neighborhoods in general [16].

With regard to health care costs, increasing physical activity rates is important because inactivity was identified to be associated with considerable costs for public health care systems in general [17–21]. Across countries, the identified direct costs linked to physical inactivity were found to represent between 1% and 2.6% of total direct health care costs [22]. In addition, indirect costs of inactivity, such as productivity losses caused by early death or disabilities, were found to even exceed the direct costs of inactivity [18, 19]. Given the substantial body of research dealing with this topic, Pratt et al. ([22], p. 173) noted that the cost burden of inactivity in wealthy countries might have been adequately examined already, but that future research would be needed for a “clinically important subpopulation such as older adults”.

The overarching objective of this research is to examine how an aging population affects the economic costs of inactivity and how physical activity can assist governments in compensating rising health care costs of aging societies. The research context for this study is Germany – one of the Western countries which will be affected by an aging population in the next decades [23]. The present analysis serves three main purposes. The first purpose concerns the estimation of empirical evidence from the past. For the period 2001–2013, direct costs of inactivity are estimated for different age-groups and related to the aging society in this period. The second purpose is to extrapolate the future development of direct costs of inactivity. Specifically, direct costs of inactivity will be projected for the years 2014–2060 by considering forecasts about the aging of the German population. The third purpose represents the projection of the impact of future physical activity promotion on the development of the direct costs of inactivity. Different scenarios including fictive reductions in physical inactivity levels are estimated.

The contribution of this study to the existing literature is three-fold. First, it is one of the first studies estimating the development of direct costs of inactivity over a longer period of time. Existing studies have only estimated the costs for one specific year. Second, it provides projections about the development of future costs of inactivity in the context of an aging society and thirdly, this study aims to quantify potential economic savings which could be generated through short-term physical activity promotion and resulting reduction in inactivity levels, respectively. These projections are important for policy development in light of an aging society.

## Methods

### Data sources

The first purpose, the estimation of inactivity costs for the period 2001–2013, requires information about the annual direct health costs and annual inactivity rates. The Federal Statistical Office [24] provided annual direct per capita health costs of several diseases differentiated by gender-specific age-groups (15–29, 30–44, 45–64, and 65+) for the years 2002, 2004, 2006, and 2008. Inflation was considered and all expenditures were converted into 2008 prices. Cost data before or after 2002–2008 were not available. Therefore, this study used an average of these four years taking into account random cost drivers (e.g. medicine prices, technological innovation) for every year. The cost variable only contains information about direct costs of diseases, including curative treatment, prevention, rehabilitation, and continuing caring.

Information about levels of physical inactivity in Germany stems from the German Socio-Economic Panel (GSOEP), a panel survey with annual measurements from 1984 to 2013 [25]. This survey has been used in previous studies to study outcomes and determinants of physical activity in Germany [26, 27]. Respondents were asked to self-report how often they engage in physical activity. Self-reported measures are usually applied in studies estimating costs of inactivity which rely on official survey datasets since large and representative sample sizes are required [17–19]. Previous research has produced mixed results on the validity of self-reported physical activity measures compared to objective ones. Whereas, for example, some studies found no significant differences between self-reported and objective measures [28], others provided evidence of an imprecise estimation due to the problem of social desirability and social approval [29, 30]. For this study, it is important to note that those potential response biases would lead to rather conservative estimates as physical inactivity levels are likely underestimated.

Depending on the respective wave, respondents could answer on a four- or five-point scale. Lechner [27] stated that both scales can be considered sufficiently similar in terms of the respective descriptive statistics. To obtain the annual rate of inactivity, the variable was then dichotomized with using 1 when the respondent was never active (this category was identical in all waves) and 0 for the other categories. Since the activity question was not included in every wave, the analysis was conducted for the years 2001, 2003, 2005, 2007, 2009, 2011, and 2013. The final sample included *n* = 166,309 observations from *n* = 41,991 individuals. More than half of the respondents were female (52.4%). The average age was 49.0 years with a range of 16–103 years. Certain population subgroups (migrants, Eastern Germans) are intentionally overrepresented in the survey. Consequently, specific sample weights provided in the dataset were used to ensure its representativeness of the overall German population [31].

The second and third purpose require predictions about future demographic changes of the German population. The Federal Statistical Office [23] has published information and projections about past and future population changes in gender-specific age-groups for the years 2014–2060. These predictions are based on a constant fertility rate of 1.4, life expectancy of 86.7 for men and 90.4 for women, and annual net migration of 200,000.

### Procedure

This section presents the analysis strategy in the order of purposes. For the first purpose, the prevalence based comparative risk assessment method developed by the World Health Organization (WHO) [32] was applied which is the common method to estimate economics costs of inactivity [22]. This method consists of the following five steps [19]:

1. Identification of diseases related to physical inactivity including their relative risks
2. Identification of the annual direct costs of each disease
3. Determination of the prevalence of physical inactivity in Germany (2001–2013)
4. Calculation of the population attributable fractions (PAF) for each disease
5. Multiplying the PAFs with the annual direct costs of each disease to determine annual direct cost of physical inactivity.

Regarding the first step, the WHO [32] stated that ischemic heart disease, ischemic stroke, colon and female breast cancer, and Type 2 diabetes were the most relevant diseases in relation to physical inactivity. In a meta-analysis, the WHO [32] determined adjusted and unadjusted relative risks (RR) for every listed disease (Table 1) which describe the ratio of the incidence rate among individuals of different age-groups and gender with a given risk factor (physical inactivity) compared to the incidence rate among individuals without it. For other socio-demographic characteristics differentiated RR are not available. This study uses the adjusted RR measures as they control for the effects of confounding factors, such as age, sex, and smoking. Since the variation between age-groups is essential to the present study, more recent RR estimates of, for example, Janssen [33] could not be used. However, the estimated confidence intervals of the RR by the WHO [32] are considered in the sensitivity analyses explained later and, hence, take into account differences to more recent RR estimates.

**Table 1:** Relative risks per disease differentiated by age (WHO, 2004)

	Age-group			
Disease (ICD-10 code)	15–29	30–44	45–64	65+
Ischemic heart disease (I20-I25)	1.47 (1.39–1.56)	1.47 (1.39–1.56)	1.47 (1.39–1.56)	1.34 (1.26–1.42)
Diabetes Type 2 (E11)	1.31 (1.24–1.39)	1.31 (1.24–1.39)	1.31 (1.24–1.39)	1.22 (1.24–1.39)
Female breast cancer (C50)	1.13 (1.04–1.22)	1.13 (1.04–1.22)	1.13 (1.04–1.22)	1.09 (1.01–1.18)
Ischemic stroke (I63)	1.39 (1.24–1.56)	1.39 (1.24–1.56)	1.39 (1.24–1.56)	1.28 (1.14–1.44)
Colon cancer (C18)	1.43 (1.38–1.49)	1.43 (1.38–1.49)	1.43 (1.38–1.49)	1.31 (1.26–1.36)

Note. *ICD = International Classification of Disease*; Upper and lower bound values in parentheses

Table 2 presents the average direct per capita costs of the five diseases mainly associated with physical activity (in 2008€) required for the second step [24]. The highest direct per capita costs can be observed for ischemic heart disease followed by Type 2 diabetes. Overall, the direct costs associated with the five diseases represent 7.9% of total direct health care costs of Germany in 2008. Ischemic heart diseases, which have the highest relative risk for physical inactivity, are alone responsible for 2.6% of the overall direct health care costs. For all five diseases the economic burden is highest for the oldest age-group 65+ years.

**Table 2:** Average direct per capita costs per disease differentiated by age (2002–2008; in 2008€; Federal Statistical Office, 2010)

	Age-groups				Total
Disease (ICD-10 code)	15–29	30–44	45–64	65+	
Ischemic heart disease (I20-I25)	0.79	11.00	91.99	282.49	96.57
Diabetes Type 2 (E11)	7.94	16.96	80.91	226.42	83.06
Ischemic stroke (I63)	0.78	3.94	24.02	139.61	42.09
Female breast cancer (C50)	0.40	9.36	37.10	52.95	24.96
Colon cancer (C18)	0.18	1.37	12.05	48.91	15.63

Note. *ICD = International Classification of Disease*

For the third step, inactivity rates of the German population were estimated (Table 3) using the data provided by the GSOEP [25]. The results revealed a notable decline in physical inactivity over the examined time-period (2001–2013). In 2001, 47.5% of males and 54.1% of females aged between 15 and 103 were physically inactive with the prevalence of inactivity generally increasing with age. Only 28.0% of people in the age-group 15–29 years were inactive compared to 79.2% in the age-group 65+ years. In 2013, inactivity rates decreased to 32.7% among males and 32.6% among females, respectively. The prevalence of inactive people regressed in particular in the oldest age-group where only 47.0% were inactive in 2013.

**Table 3:** Inactivity rates of the German population, 2001 and 2013 (in %; GSOEP, 2014)

	Inactivity rate in 2001		Inactivity rate in 2013	
Age-group	Male	Female	Male	Female
15–29	24.5	32.4	18.1	15.9
30–44	36.1	39.8	23.7	23.3
45–64	54.5	55.5	33.3	31.4
65+	75.7	81.6	45.2	48.6
Total	47.5	54.1	32.7	32.6

In a fourth step, by using the rates of inactivity and the RR of each disease, PAFs for each disease were calculated. A PAF identifies the proportion of a disease which can be associated with a certain risk factor (here: physical inactivity). It is estimated with the following equation:

1 $$ \mathrm{PAF}=\left[\mathrm{P}\left(\mathrm{RR}\hbox{--} 1\right)\right]/\left[1+\mathrm{P}\left(\mathrm{RR}\hbox{--} 1\right)\right] $$

where P denotes the prevalence of physical inactivity and RR the relative risk for a disease if someone is inactive (Table 1). The inactivity rates were estimated for gender-specific age-groups for the years 2001–2013. In a final step, the resulting PAFs were multiplied by the annual total direct costs for each disease to estimate annual direct costs of inactivity differentiated by gender and age-groups.

Considering the second purpose, the direct per capita inactivity costs of each disease for every gender-specific age-group from the year 2013 were multiplied by projected age- and gender-specific population numbers for the years 2014–2060. The annual total direct costs per disease were then aggregated to estimate total direct costs of physical inactivity for every year. This method has been previously applied to forecast the impact of aging on health costs [6, 34].

The third research purpose builds on the projections of the second purpose, but considers two fictive scenarios predicting how future short-term actions to promote physical activity could impact projected direct costs of inactivity. The first scenario assumes that the inactivity rates in all age-groups could be reduced by five percentage points until 2020. The authors decided to use five percentage points because this value reflects the observed negative compound annual growth rate of physical inactivity of 3.6% for the period 2001–2013. Moreover, this figure has already been used in previous studies to estimate the impact of fictive physical activity reduction targets [35, 36]. The second scenario assumes that only the inactivity rate in the age-group 65+ years can be reduced by five percentage points until 2020, which is reasonable, since the level of inactivity in all other groups already finds itself on a relatively low level compared to other countries [10]. Also, the German government prioritized healthy aging including promotion of physical activity for the elderly as one of seven national health goals [37]. For both scenarios, new PAFs were calculated and direct per capita costs of inactivity for the year 2020 were estimated. Afterwards, the projection procedure described in the second purpose is repeated for the period 2020–2060.

### Sensitivity analysis

Since all three purposes are based on calculations using numerous input factors, this study conducted sensitivity analyses considering variations among those determining factors. Therefore, in line with Ding et al. [38], an extreme scenario approach was used. When conducting an extreme scenario sensitivity analysis, every factor is set at its minimum and maximum value to generate a lower and upper bound of the analyses. The relevant input factors for the first research purpose were costs of the different diseases and relative risks. Regarding disease-specific costs, we followed Katzmarzyk et al. [39] and varied each disease-specific health care cost by ±20%. For the relative risks, the confidence interval estimated by the WHO [32] was used. For the second research purpose, in addition to health costs and relative risks, the lower and upper bound of the projected development of the German population was used as reported by the German Federal Statistical Office [40]. The lower bound includes a fertility rate of 1.4, an average life expectancy of 84.8 for men and 88.8 for women, and a net migration of 100,000. The upper bound is based on a fertility rate of 1.6, an average life expectancy of 86.7 for men and 90.4 for women, and a net migration of 200,000. The estimated scenarios of the third research purpose relied on the same variations in costs, relative risks, and population projections.

## Results

**Fig. 1:**
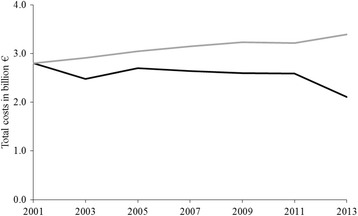
Costs of inactivity, 2001–2013 (in 2008€). Development of inactivity costs from 2001 to 2013 (black line). In comparison the development of inactivity costs if the level of inactivity among the German population had remained constant since 2001 (grey line)

The results related to the first purpose of providing evidence from the past are visualized in Fig. 1. They reveal that the overall direct costs of inactivity amounted to €2.80 billion and €40.13 per capita in 2001, respectively, which represents 1.3% of the total direct health care costs of people older than 15 years. The sensitivity analyses revealed a lower bound of inactivity costs in 2001 of €1.70 billion and an upper bound of €4.16 billion.

During the 13-year observation period, the costs of inactivity have declined almost constantly. In 2013, the overall direct cost were reduced by 24.7% to €2.11 billion and €30.10 per capita, respectively, which represents a negative compound annual growth rate of 2.3%. Consequently, the proportion of total direct health care costs was reduced to 0.9%. For 2013, the best-case scenario indicated costs of €1.26 billion and the worst-case scenario costs of €3.19 billion.

Figure 1 also shows how the direct costs would have developed if the rate of inactivity remained constant since 2001. In this case, the inactivity costs would have increased by 21.0% to €3.39 billion, which represents a compound annual growth rate of 1.6% and is 60.7% higher than the actual costs of inactivity in 2013.

For the second purpose of projecting the effects of aging on the costs of inactivity for the years 2014–2060, the predictions of the population development provided by the Federal Statistical Office [23] indicate that the average age of the German population will increase from 44.2 years in 2013 to 49.7 in 2060 [23]. This development will mainly be driven by an increase in people who are 65+ years old. In 2013, 21.7% of all people older than 15 years were aged over 65 years. In 2060, this share is expected to increase to 30.2%.

**Fig. 2:**
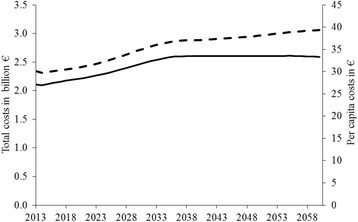
Costs of inactivity, 2014–2060 (in 2008€). Predicted costs of inactivity for the period 2014–2060 if physical inactivity remains on the level of 2013. Total costs (solid line) are compared to per capita costs (broken line)

Figure 2 presents the direct costs of inactivity associated with the predicted aging of the German population. First, the overall annual direct costs of inactivity will rise by 22.9% from €2.11 billion in 2013 to 2.60 billion in 2039 (in 2008 €). Afterwards, a plateau will be reached and annual direct costs will remain constant, amounting to €2.59 billion in 2060. However, direct per capita costs are predicted to grow continuously. They increase to their maximum value of €39.03 in 2060 which represents an overall increase of 30.7% from 2013 with a compound annual growth rate of 0.6%. The extreme scenario sensitivity analysis shows that the plateau of total cost of inactivity in 2039 can be between €1.49 billion (lower bound) and €3.93 billion (upper bound). The increase in per capita costs until 2060 can vary between €23.21 (lower bound) and €57.89 (upper bound).

**Fig. 3:**
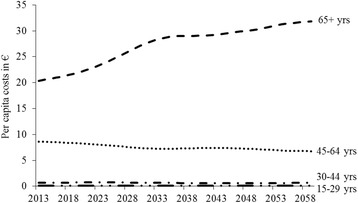
Per capita costs of inactivity by age-group, 2014–2060 (in 2008€). Predicted per capita costs of inactivity for the period 2014–2060 differentiated by four age groups (15–29; 30–44; 45–64; 65+)

Figure 3 provides a more detailed analysis of per capita costs of inactivity by age-group. It indicates that for the age-groups 15–29 years and 30–44 years the per capita costs remain almost constant over the examined period, while the per capita costs for the age-group 45–64 even slightly decrease from €8.81 in 2013 to €6.79 in 2060. The only increase can be observed for the oldest age-group of 65+ years. Within this age-group, per capita costs increase from €20.54 in 2013 to €31.83 in 2060 which represents an overall increase of 54.9% and a compound annual growth rate of 0.9%.

The results of the third purpose analyzing two scenarios with fictive short-term targets of reducing physical inactivity are displayed in Table 4. Recall that the first scenario comprised a reduction of physical inactivity among all age-groups by five percentage points until 2020. Applying this scenario, the resulting accumulated direct costs of inactivity would amount to €104.5 billion at the end of 2060. This would represent an 11.2% decrease of the original predicted values for 2060 based on the inactivity level of 2013. Overall this could lead to savings of €11.70 billion until 2060 representing annual cost savings of €292.48 million. The second scenario assumed that only the inactivity level in the oldest age-group (65+ years), the most inactive one, will drop by five percentage points. The results for this scenario show that 73.6% of the cost savings of the first scenario could be achieved which amounts to total savings of 8.60 billion and annual cost savings of €215.17 million from 2020 to 2060.

**Table 4:** Accumulated projected costs of inactivity, 2020–2060 (in billion 2008€)

Scenario	Years				
	2020	2030	2040	2050	2060
Inactivity rate remains at 2013 level	15.07 (8.99–22.76)	38.38 (22.70–58.03)	64.07 (37.47–96.97)	90.12 (52.05–136.46)	116.15 (66.13–175.98)
5 pp. reduction in inactivity rate of all age-groups	15.07 (8.99–22.76)	35.61 (20.99–54.02)	58.34 (33.98–88.66)	81.39 (46.80–123.81)	104.45 (59.20–159.02)
5 pp. reduction in inactivity rate of age-group 65+	15.07 (8.99–22.76)	36.46 (21.53–55.23)	59.97 (35.00–90.99)	83.79 (48.29–127.24)	107.54 (61.11–163.46)

*Note:* Upper and lower bound values in parentheses

## Discussion

This study investigated the influence of physical inactivity – a major risk factor for numerous diseases – on the development of health care costs in Germany in the light of an aging population. It had three different research purposes focusing on (1) evidence from the past, (2) the prediction of future costs of inactivity, and (3) the effect of future physical activity promotion. Regarding evidence from the past, 1.3% of total direct health costs could be attributed to physical inactivity in 2001. This finding is in line with previous research from several countries, including the UK, Canada, and the United States [17, 39, 41] where estimated costs of inactivity ranged between 1% and 2.6% on average [22]. A comparison with studies estimating the economic burden of obesity and smoking on the German health care system to be at 2.1% [42] and 3.3% [43], respectively, indicates that physical inactivity can be considered a notable public health burden for Germany.

The costs of inactivity have significantly declined over the 13-year timespan. This development was driven by an overall decrease in inactivity rates in Germany. At least two explanations can be advanced for this finding. First, the results may indicate a rising awareness of the German population for the individual benefits of physical activity [44], potentially leading to higher activity rates. Second, during the same period, the German government launched several initiatives to promote physical activity, especially among the elderly [45, 46], which may have contributed to increasing activity rates as well. Over this time-span, the average age has increased from 41.3 years in 2001 to 44.2 years in 2013 [40]. Moreover, the proportion of people aged over 65 years increased from 20.2% to 24.0% of all people older than 15 years. Consequently, if the inactivity rate had remained at the level of 2001 for this period, the PAFs would have been held constant, meaning that a resulting 21.0% increase in costs could have been exclusively attributed to the aging of the German population during this period. Hence, the negative effect of aging on costs of inactivity was compensated by an increase in physical activity patterns among the German population during the period 2001–2013.

Turning to the second purpose, the projected progression of future total costs of inactivity for the period 2014–2060 shows a plateau to be reached in 2039. One explanation for the following almost constant annual direct costs might be that at the end of the 2030s most of the *German baby boomers* [47] – born between 1955 and 1969 – will have passed away and, hence, the overall population will shrink which compensates the increases in direct health costs related to aging. However, the continuous increase in direct per capita costs during this period indicates that the economic burden for the German population will not decline. The analysis by age-groups revealed that the increase in direct costs of inactivity can almost solely be attributed to the age-group 65+ years. As a consequence, although the effect of aging on direct costs of inactivity was compensated by a considerable decrease in inactivity rates in Germany for the period 2001–2013, over the next decades aging will eat up 92.2% of the gains accomplished during the period. Since the possibility of a decline in physical activity has to be taken into account, the economic burden could even increase if past patterns of physical inactivity reoccur. Hence, the ongoing aging of the German population demands further policy initiatives.

The third purpose of this study was to estimate the effect of those potential initiatives to reduce physical inactivity on direct costs of inactivity. The results stress the economic importance of promoting physical activity in particular among the elderly as a decrease in physical inactivity by 5 percentage points only in the oldest age-group of 65+ years can generate 73.6% of the costs savings if physical inactivity is reduced by 5 percentage points among all age-groups.

Concerning policy development, it is important to consider that policies aimed at reducing physical inactivity bear different effects for socio-demographic groups. For example, Humphreys and Ruseski [48] revealed that spending on parks and recreation only has a significant effect on outdoor physical activities preferred by younger people with higher education and higher income. Wicker et al. [15] showed that the effect of different types of sport infrastructure varies between age-specific target groups and Downward and Rasciute [49] documented that satisfaction with sport facility provision had a significant positive effect on health related activity levels of females, but not of males. Consequently, governments have to be aware of those differences if they want to target specific socio-demographic groups, such as the elderly.

## Conclusion

The results of this study add to the existing literature in at least three ways. First, it is one of the first to analyze direct costs of inactivity over a longer period of time using panel data, enabling insights how costs are influenced by changing physical activity patterns of a population and demographic changes (e.g. aging society, shrinking population). Previous research has only used cross-sectional data and estimated the economic costs of inactivity for one year [17, 39, 41]. Second, it provides a more detailed analysis by differentiating between four age-group and both sexes. Such a detailed analysis takes into account that the most important parameters for estimating costs of inactivity, i.e., inactivity rates and relative risks, vary by age and gender. Third, it contributes to the body of research by providing insights about the role of physical inactivity in the context of aging and development of health care costs; previous research has only looked at other risk factors, such as smoking and obesity [7, 50].

The findings come with implications for policy makers and public health organizations. Overall the results indicate that the promotion of physical activity can be used as an important non-pharmaceutical action to reduce public health care costs considerably. Moreover, especially increasing physical activity rates among the elderly can assist governments in compensating the increasing demand for health care resulting from an aging population. In addition, the findings outline the urgency of short-term actions because a – well feasible – reduction of physical inactivity within the next five years could lead to long-term economic benefits for the national health care system. Since approximately 75% of cost savings can be attributed to the oldest age-group of 65 + years, initiatives and campaigns promoting physical activity should particularly target this age-group. Considering that costs occur for both designing and launching such campaigns and initiatives and their efficiency can differ significantly between age-groups [51], promotion campaigns for older age-groups ought to be prioritized. Such policy priorities are to be deemed especially important because public authorities are faced with budget constraints.

This study has some limitations which could guide future research. First, it is necessary to note that the population projections made by the Federal Statistical Office [23] only consider the aspect of aging, while possible changes in technology or behavior, and health policy reforms have not been taken into account. Furthermore, existing research suggests that not only age effects are important when investigating physical activity patterns over time, but also period and cohort effects [52], which represent an avenue for future research. Finally, since this study only uses a measure of the frequency of physical activity, future research would benefit from more detailed measures also considering activity duration and intensity that allow calculating metabolic equivalents (METs).

## Abbreviations

GDP: Gross domestic product
GSOEP: German Socio-Economic Panel
PAF: Population attributable fraction
RR: Relative Risks
WHO: World Health Organization
